# Celebrating the 25 years of the Journal of Applied Oral Science

**DOI:** 10.1590/1678-77572017ed001

**Published:** 2017

**Authors:** Karin Hermana Neppelenbroek, Vanessa Soares Lara

We proudly announce that in 2017 the Journal of Applied Oral Science (JAOS) is celebrating 25 years of existence. As an official nonprofit publication of the Bauru School of Dentistry (FOB), University of São Paulo (USP), this achievement deserves our respect and acknowledgements to all the authors that chose our journal to publish their contributions in the areas of Dentistry and Speech-Language Pathology and Audiology as well as related areas. The anonymous work of the reviewers and associate editors was also instrumental for the quality of the works published in the JAOS, which was translated into the indexation of the journal in internationally respected databases such as PubMed/MEDLINE, PubMed Central^®^ (PMC), Web of Science, Journal Citation Reports^®^ (JCR), SCOPUS and Scientific Electronic Library Online (SciELO).

Great part of JAOS's success results from a dedicated work of all previous editors including those that started the former title “Revista da Faculdade de Odontologia de Bauru”. Therefore, this is the perfect time to acknowledge them and remember their names: Aguinaldo Campos Junior, Rumio Taga, José Mauro Granjeiro, Ricardo Marins de Carvalho, Carlos Ferreira Santos and Gustavo Pompermaier Garlet. We assure these colleagues that we are doing our best to keep the invaluable work they devoted to our journal.

Once again we thank all the employees involved in the JAOS's publication: Valéria Cristina Trindade Ferraz, José Roberto Plácido Amadei, Deborah Schmidt Capella Junqueira, Sônia Cláudia Antonelli Pirola, Neimar Vitor Pavarini, Rubens Kazuo Kato, Allan Rodrigo Dias, Camila Medina and Zelma Batista Borges.

Finally, we express our gratitude for the financial support provided over the last 25 years by the Rectory of USP, the Deans of FOB, the National Council for Scientific and Technological Development (CNPq), the Coordination for the Improvement of Higher Education Personnel (CAPES) and the State of São Paulo Research Foundation (FAPESP).

Karin Hermana Neppelenbroek Editor-in-Chief Vanessa Soares Lara Co-Editor-in-Chief

